# Three‐dimensional surface scanning of patients to extend computed tomography field of view and scan length for dose calculation and collision avoidance in radiotherapy treatment planning

**DOI:** 10.1002/acm2.70333

**Published:** 2025-11-10

**Authors:** Amanda M. Jackson, Deepak K. Shrestha, Perry B. Johnson

**Affiliations:** ^1^ Department of Radiation Oncology WashU Medicine, 4921 Parkview Place St. Louis Missouri USA; ^2^ Department of Radiation Oncology Mayo Clinic Jacksonville Florida USA; ^3^ Department of Radiation Oncology University of Florida College of Medicine Gainesville Florida USA; ^4^ UF Health Proton Therapy Institute Jacksonville Florida USA

**Keywords:** 3D scanning, 3D surface, ALARA, collision detection, FOV limitations, SGRT, surface alignment

## Abstract

**Background:**

Radiotherapy treatment planning requires knowledge of a patient's anatomical surface for use in dose calculations, collision checks, or surface‐guided radiation therapy (SGRT). This information is usually obtained from a computed tomography (CT) scan which may encounter field‐of‐view (FOV) limitations or require additional radiation dose beyond the treatment area to capture the full patient habitus.

**Purpose:**

This study demonstrates an affordable alternative solution to obtain a patient's surface information without CT or room mounted surface‐guidance imaging cameras for the purpose of detecting patient collisions and providing additional skin surface information needed for appropriate depth calculations of clipped FOV scans.

**Methods:**

Three‐dimensional (3D) surface models of five patients were acquired immediately prior to CT simulation using an iPad Air 2 equipped with a Structure Sensor Pro and Monocle SS application. The 3D surfaces were manually registered to the CT images and nearest distances between CT surface and 3D surface were calculated. Nearest distance calculations were also performed with a 2 mm CT rind structure that excluded select regions not relevant to surface detection. To demonstrate the advantage of having additional scan length for clearance checks, a collision simulated plan was evaluated with a shortened CT scan length versus with additional 3D surface information.

**Results:**

CT rind to 3D scan surface mean distances for each patient were 1.65 mm, 0.99 mm, −0.93 mm, −0.79 mm, and −1.53 mm with standard deviations of 6.51 mm, 6.71 mm, 3.38 mm, 2.84 mm, and 2.69 mm.

**Conclusion:**

This study demonstrates the feasibility of an accessible low‐cost 3D surface scanning solution to supplement clipped field‐of‐view CT scans and reduce CT scan lengths by obtaining important patient surface data for improved calculation accuracy, treatment clearance checks, and SGRT.

## 1 INTRODUCTION

Radiotherapy treatment planning requires computed tomography (CT) scans for anatomical and relative electron density information to model the radiation dose distribution within the patient. Due to the bore size of the CT scanner and a desire to minimize radiation dose outside the region of interest, a CT scan of a patient may have a limited field of view (FOV) or restricted scan length that does not fully capture their entire anatomy. For example, a patient with a larger body habitus may have portions of their anatomy excluded from the scan axially, potentially impacting the accuracy of the dose calculation. Similarly, a limited scan length may inhibit virtual clearance checks that can be used to identify potential collisions between the patient and the treatment machine. A limited FOV may also affect the accuracy of surface‐guided radiation therapy (SGRT), which relies upon matching the patient's surface with a reference surface derived from their CT scan.

Some of these issues can be addressed by extending the CT scan length, particularly to capture details about the arm or head positions. However, this results in additional imaging dose to the patient. Concerns for minimizing radiation exposure to be as low as reasonably achievable (ALARA) have been expressed over many decades by multiple organizations^1^ such as the National Council on Radiation Protection and Measurements (NCRP),^2^ the Nuclear Regulatory Commissiong (NRC),^3^ and the Centers for Disease Control (CDC).^4^ Additionally, physicians agree on the importance of balancing the benefits of additional CT imaging with the potential radiation risks from imaging dose,^5^ particularly if a patient is under 40 years of age.^6^

Instead of relying solely on additional CT imaging, three‐dimensional (3D) surface modeling provides an alternative means to capture missing anatomical details, offering valuable supplementary data for treatment planning and patient setup. There are several techniques available to obtain a 3D surface model of the patient. One technology commonly used in radiation oncology departments acquires 3D patient surface models at the time of CT simulation using video‐based stereo photogrammetry.^7^ In this approach, a speckled optical pattern is projected onto the patient's skin using ceiling‐mounted camera units. Image sensors capture the distortion of this textured pattern, and a 3D surface rendering of the patient is reconstructed from the images. This technology combines two commonly used three‐dimensional mapping methods: stereo vision and structured light.^8^, ^9^, ^10^ Stereo vision mapping involves dual cameras that function similarly to human eyes, allowing depth perception by correlating detected features from different viewpoints. Structured light mapping detects distortions in a projected textured pattern and uses them to calculate the object's surface shape. Both methods contribute to the principles of photogrammetry, which identifies points, patterns, or features from captured images and maps them to generate an accurate surface representation of the object.^11^

The existing commercial method for surface mapping uses fixed cameras and can be costly and impractical for some clinics. A full platform which extends to the treatment machine for use with patient setup could cost a clinic hundreds of thousands of dollars, making it a significant investment. Several patient surface mapping tools, using a laser scanning system, handheld stereo depth camera, handheld LiDAR scanner, and infrared scanner have been evaluated. All of them demonstrate agreement within 1.5 cm between the CT‐derived surface and the corresponding surface models.^12^, ^13^, ^14^, ^15^ Such studies highlight the opportunity for a more economical option for clinics that only require additional patient surface data to aid in treatment planning and collision avoidance. The work presented in this manuscript builds on these concepts and further demonstrates an accessible method to acquire patient surface models by using tools that may already be present in radiation oncology departments or can be easily acquired.

## 2 METHODS

### 2.1 Supplementing a CT dataset with a 3D surface model

The surface scanning tool, Decimal 3D (.decimal, Sanford, FL), was introduced in radiation oncology workplaces in 2020 to perform electron treatment simulations without a CT scan or time on a linear accelerator to determine the treatment field geometry. The tool consists of an iPad (Apple, Cupertino, CA) equipped with a Structure Sensor (Structure, Boulder, CO) that is used with the Decimal 3D application to map a patient's skin surface for planning of electron treatments. The manufacturer of the Structure Sensor provides limited information about the technology it uses, but prior publications suggest it is based on infrared depth‐sensing, which utilizes structured light mapping, projecting a focused, non‐uniform pattern of infrared dots.^16^ The original Structure Sensor Pro model uses 825 nm operating wavelength.^17^ The recent Structure Sensor 3 model uses 940 nm operating wavelength and boasts 1 mm accuracy with 33 cm to 20 m of range.^18^

**FIGURE 1:**
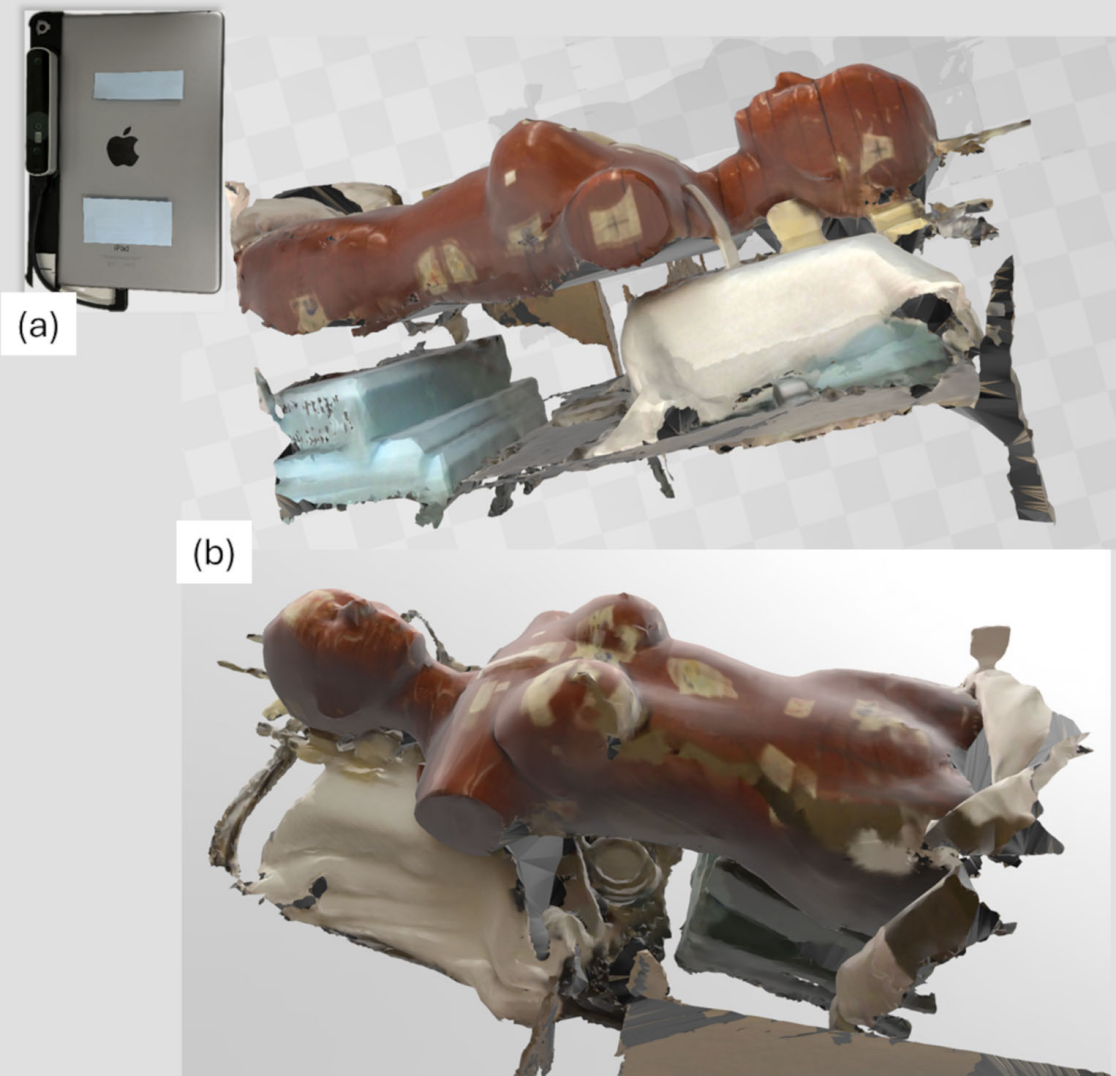
3D surface image of RANDO phantom. (a) The surface was acquired with an iPad Air 2 and Structure Sensor Pro. (b) The resulting surface image of the phantom is shown.

An iPad Air 2 equipped with Structure Sensor Pro was available for this study from previous use of the Decimal 3D application. To repurpose this hardware and explore its capabilities, the free Monocle SS (v3.5.0, Steampunk Digital, Co., Fukuoka, Japan) application was installed on the iPad to test the system's ability to capture surface area. When running the application, the iPad and sensor must be moved around the object of focus until all the desired surfaces are visually shown in the application surface map. A test was conducted on a RANDO phantom with results shown in Figure 1. By default, the system also captures all objects on which the phantom is resting, as seen in the images.

The 3D surface model was exported from the Monocle application as filetype .obj and processed with the freely available application, 3D Builder (v20.0.4.0, Microsoft, Redmond, WA), to specify a meter size scaling of the file. Also freely available, 3D Slicer (v5.2.2, Brigham and Women's Hospital, Harvard Medical School, Boston, MA^19^, ^20^) was utilized to assign patient orientation and convert the .obj file to a binary labelmap. A binary labelmap assigns either 1 or 0 value to the voxels of the dataset, where the surface model is assigned a label of 1 and the rest of the image is assigned a label of 0. The processed file was then exported as a DICOM image set and the label value of 1 became Hounsfield Unit (HU) equal to 1. The newly created DICOM image set was imported into the Varian Eclipse treatment planning system (v15.6), where it was manually registered with the CT simulation image set. The 3D surface image set was then segmented using thresholding of 1 HU, creating a surface structure which could be visualized to aid registration with the CT simulation images.

To incorporate the 3D surface model in treatment planning, it needs to be added to the treatment planning CT image. The surface structure can be copied over to the patient's CT structure set via the registration that was manually applied. However, if this surface is used to extend the dataset, it will be clipped by the CT scan length. To prevent this, the patient dataset length must be extended to fully accommodate the full 3D surface into the CT structure set. If desired, a density override can be applied but is only needed for dose calculation purposes.

### 2.2 Clinical Trial

**FIGURE 2:**
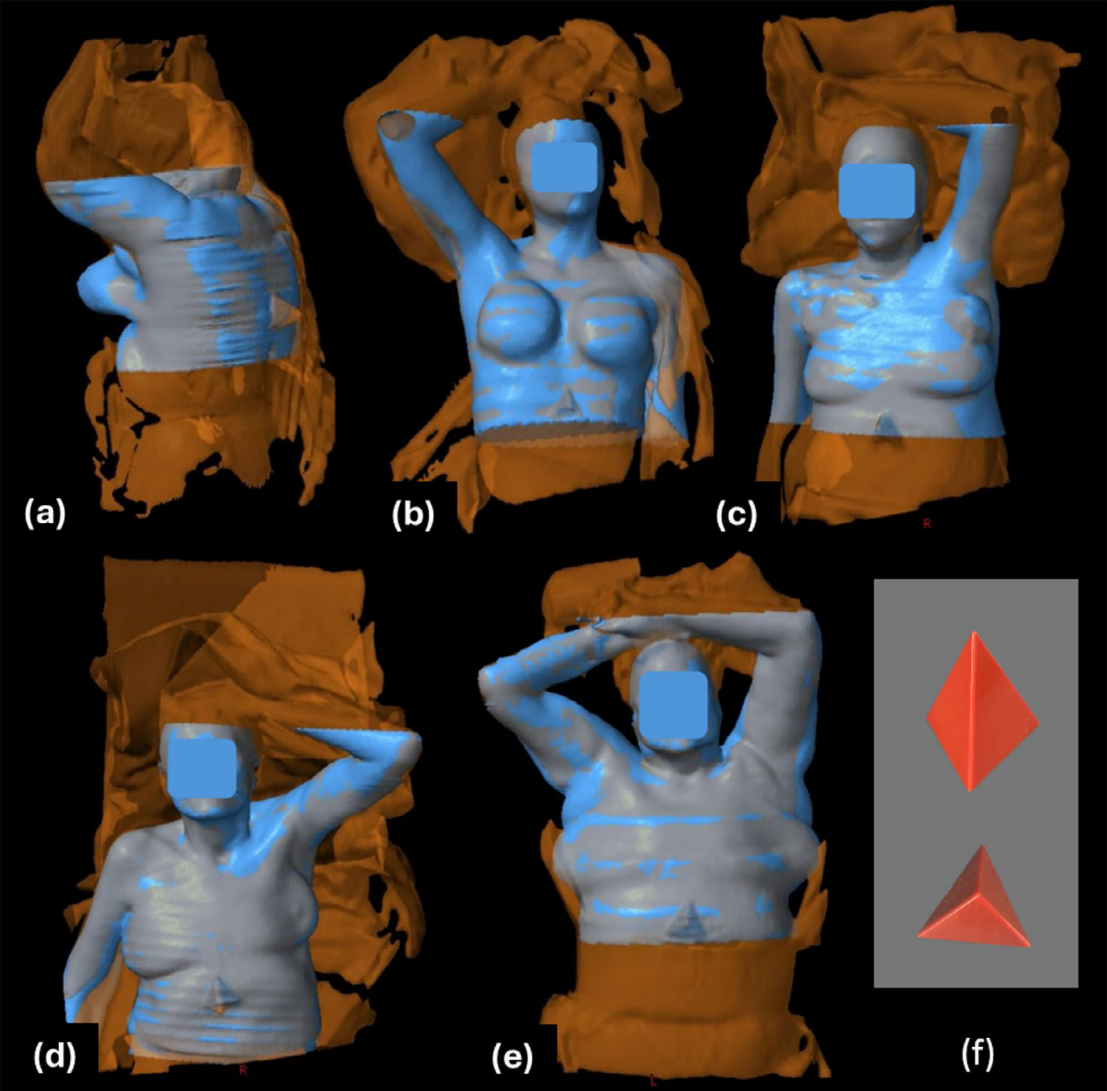
Overlay of CT image surface (blue) and 3D scan surface (orange). (a) Patient 1, (b) Patient 2, (c) Patient 3, (d) Patient 4, (e) Patient 5, (f) plastic pyramid patient marker. The 3D scan surface shows noticeably more information, including clipped arms and the patient immobilization.

A small patient trial was established with Institutional Review Board (IRB) approval and an accrual goal of five patients to assess the feasibility and initial accuracy of the system directly on patients in a clinical setting. After obtaining informed consent, the patients were set up for CT simulation as ordered by the attending physician with the appropriate immobilization devices and the addition of a small plastic pyramid that was taped to the patient's skin at the edge of the scan. The pyramid (Figure 2f) serves as a quick reference to aid alignment of the acquired surface image with the CT image, particularly for patients with fewer anatomical landmarks. The 3D surface scan was taken immediately before the CT scan using the iPad Air 2 with attached Structure Sensor Pro and the iPad Monocle SS application.

After merging a patient's 3D surface structure with their CT surface in Eclipse, the DICOM structure set was exported and processed in 3D Slicer. A model to model distance computation was performed in which the distance is computed at every point of the source model to the closest proximity point in the target model. The CT scan was determined to be the sparser dataset due to the shorter scan length, and therefore the distances measured from CT to 3D were computed. The results file was analyzed with a simple Python script to determine the mean and standard deviation of the distances. Initial analysis of the full dataset demonstrated a need to exclude select regions that are not relevant to surface detection. Given the comparison is of an enclosed 3D surface, the hidden areas of the patient, such as the posterior aspect of a supine patient, do not show good agreement and are not relevant. Cropping both volumes to the same FOVs skewed the results to falsely appear better by comparing volume surfaces on cropped edges that match perfectly.

To evaluate the model to model mean and standard deviation distances for the relevant surfaces, a 2 mm thick rind structure was created from the CT surface areas of interest. Multiple cropped FOV segments were created, targeting areas of visible skin surface. Known discrepancies, such as hidden areas of the patient, regions outside the CT or 3D scanner field of views, or where sheets or immobilization were covering the patient skin, were deemed irrelevant and excluded from the evaluation field of view. The segments were Boolean summed, expanded with a 1 mm uniform margin, and then a 1 mm contracted full CT surface was Boolean subtracted from this to create the 2 mm thick rind structure. Although it introduces geometric alteration to the surface and incorporates small error, it helps reduce the gross error by removing surfaces that do not correspond to visible anatomy. Both signed and absolute differences were calculated.

### 2.3 Clearance Check Demonstration

As mentioned previously, and as one of the motivators of this study, a lack of CT scan length can cause a clearance concern to be overlooked and only discovered later during treatment. To demonstrate the benefit of having more complete surface information with additional 3D scan data, one of the trial patient's CT scan was shortened to exclude the arms and a treatment isocenter placed in a posterior and lateral position that was likely to be a concern for collision. The Radformation ClearCheck (Radformation, Inc, New York, NY) clearance check tool was utilized to evaluate treatment clearance with both the shorter CT scan and the CT scan extended with 3D scan surface data that included the arms.

## 3 RESULTS

### 3.1 Clinical Trial

**FIGURE 3:**
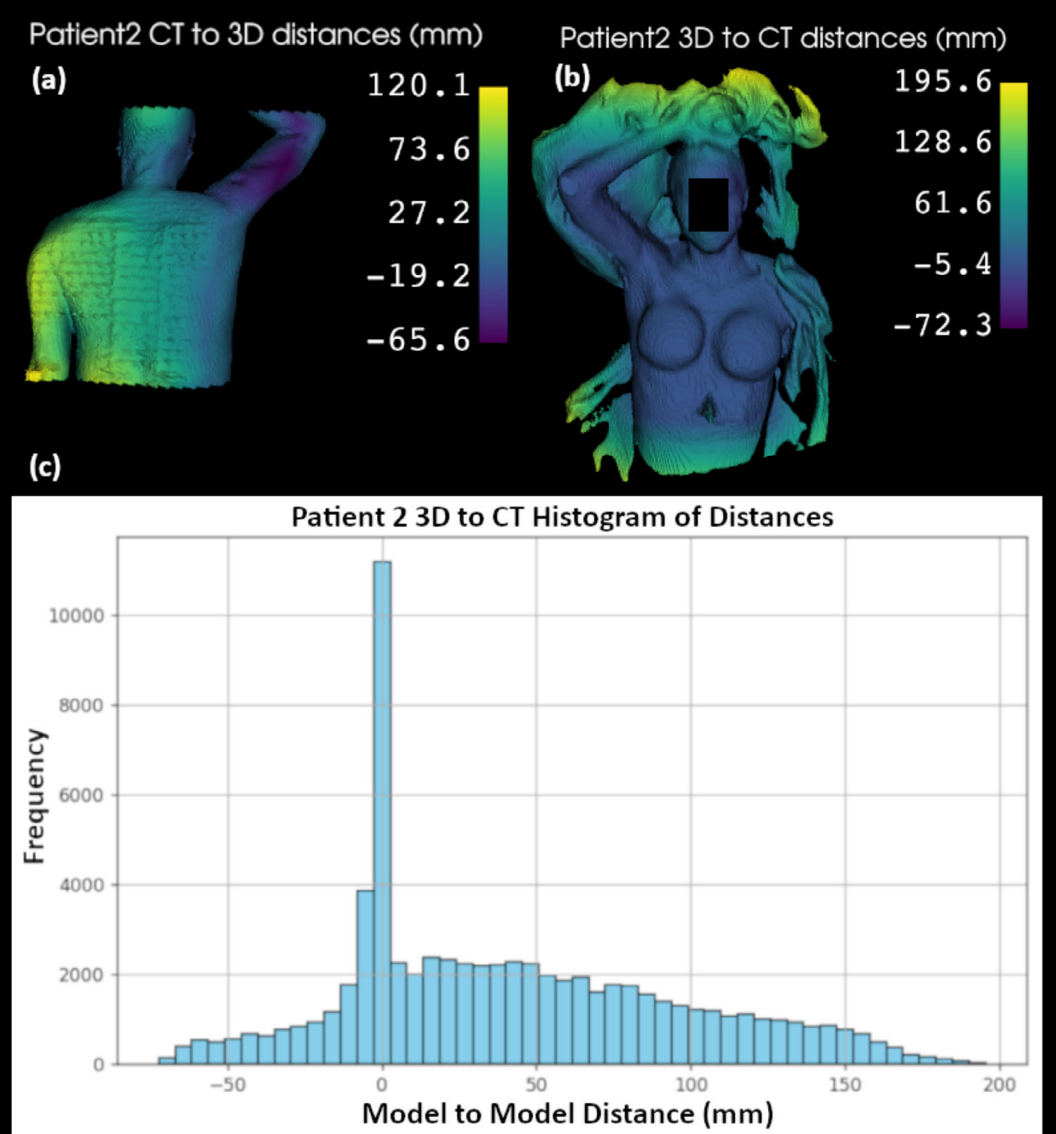
Model to model distance maps and histogram for patient 2, showing the greatest disagreement in areas where the 3D and CT FOVs differ. (a) Patient 2 CT to 3D distances showing the hidden posterior aspect of the patient that could not be 3D scanned, (b) Patient 2 3D to CT distances showing the extended data and immobilization information that does not correlate with the acquired CT surface, (c) Histogram of all the distances calculated for the 3D to CT evaluation.

Figure 2 shows an overlay of the 3D and CT surfaces. The 3D mapped surface (orange) includes both the immobilization devices and a greater patient length than the CT surface (blue). The impact of this additional patient length included in the 3D scan, along with its capture of immobilization and blankets, and the lack of data that could be captured on the hidden areas of the patient is demonstrated in Figure 3. The patient's exposed skin surface shows model to model distances closer to 0 mm, while the aforementioned areas show greater distances as there is no nearby surface to compare to. Areas at superior and inferior ends of the CT dataset, along with areas where the 3D surface volume was enclosed posteriorly, are included although not ideal as these areas are not correlated with surface data but are inside of the patient. The histogram demonstrates a large peak of model to model distances near 0 mm, further supporting the need to crop the area of data analysis.

**FIGURE 4:**
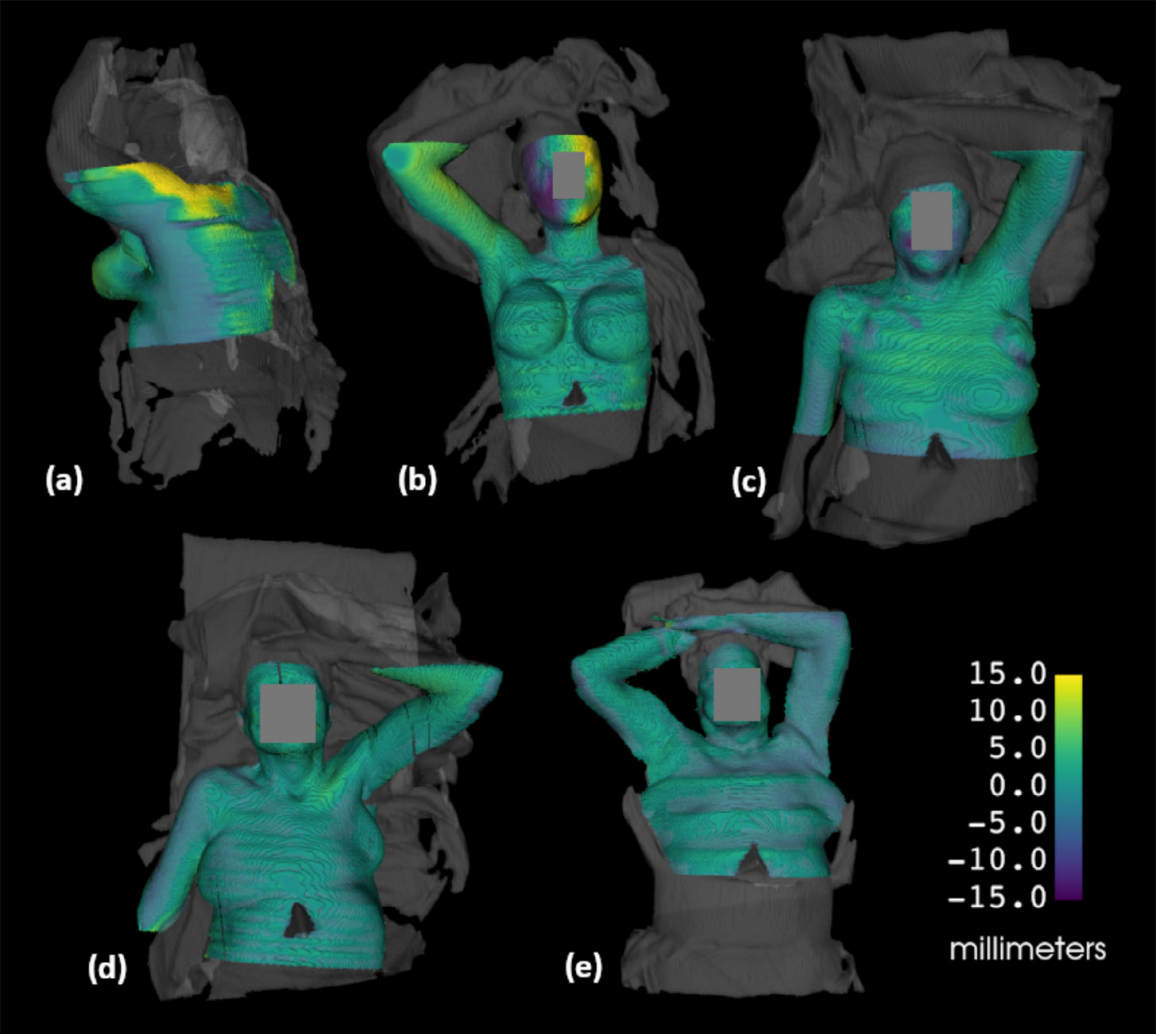
Model to model distance maps for the 2 mm CT rind to relevant 3D surface FOV, overlayed with the full 3D surface in grey. (a) Patient 1, (b) Patient 2, (c) Patient 3, (d) Patient 4, (e) Patient 5.

Model to model mean distance and standard deviation of the CT to 3D surfaces are summarized in Table 1. The following dataset evaluations are presented: full CT to 3D, CT 2 mm rind to 3D distances, and CT 2 mm rind to 3D absolute distances. The patients’ treatment sites and simulation orientations are also indicated. A visual presentation of the rind results, with color indicating distance to the 3D surface, is seen in Figure 4. A movement of the second patient's head between the 3D and CT scans was detected and is indicated by the purple and yellow colors on the side of their face (4b).

**TABLE 1:** The model to model mean distance and standard deviation calculated for all five clinical trial patients between the following datasets: full CT to 3D, CT 2 mm rind to 3D with signed values, and CT 2 mm rind to 3D with absolute values.

Model to Model Distance						
Patient		#1	#2	#3	#4	#5
Site		Left Breast	Right Breast	Left Breast	Left CW/Thorax	Bilateral Lung
Orientation		Prone	Supine	Supine	Supine	Supine
CT to 3D	Mean (mm)	−13.67	14.19	1.26	2.10	24.70
	Std Dev (mm)	31.71	31.56	21.69	31.27	44.26
CT Rind to 3D Signed	Mean (mm)	1.65	0.99	−0.93	−0.79	−1.53
	Std Dev (mm)	6.51	6.71	3.38	2.84	2.69
CT Rind to 3D Absolute	Mean (mm)	4.76	4.71	2.74	2.37	2.44
	Std Dev (mm)	4.74	4.88	2.19	1.75	1.90

### 3.2 Clearance Check Demonstration

**FIGURE 5:**
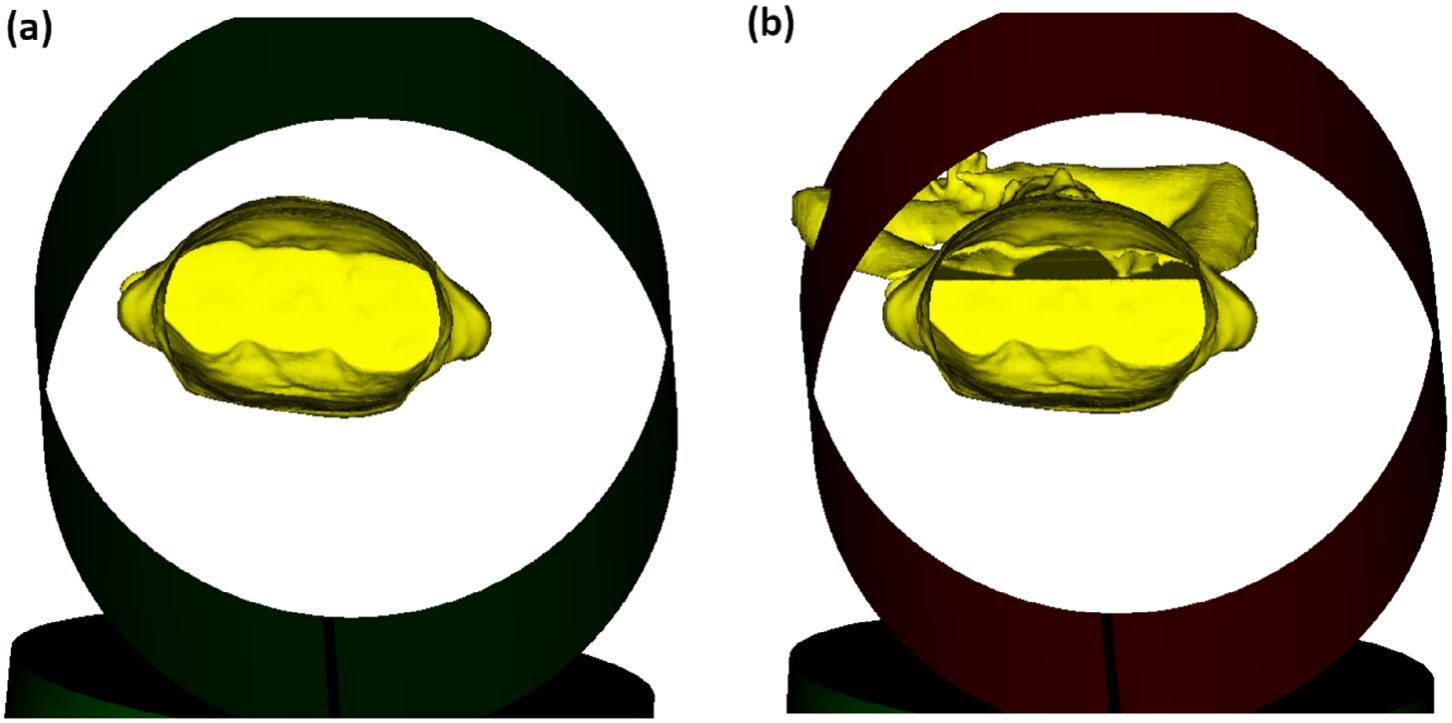
Radformation ClearCheck collision detection tool results for patient 5 setup with posterior left lung isocenter. (a) Collision evaluation with a shortened CT surface (b) Collision evaluation with the 3D surface. This demonstrates how the lack of CT scan length can cause a clearance concern to be overlooked and discovered at treatment.

A posterior left lung treatment isocenter was placed on patient five such that a collision concern was induced. Radformation ClearCheck collision detection tool was utilized to evaluate the clearance with a shortened CT scan that did not include the patient's arms and the CT scan extended with 3D scan surface data. The results in Figure 5 demonstrate a collision was not detected on the shorter CT scan (5a) but was detected using the additional 3D surface information (5b).

## 4 DISCUSSION

3D surface scanning presents an opportunity for further exploration of use in radiation oncology workflows. While SGRT has been used for over a decade at treatment, incorporating surface scanning at the time of CT simulation can provide supplemental information that the CT scan alone may not capture. 3D surface data can be used for clearance checks, a feature currently offered by some vendors typically with proprietary software platforms separate from the treatment planning system, although recent integration is being demonstrated.^21^ If methods involving 3D surface capture as present in this study are implemented, such data can be imported into the treatment planning system and used with a clinic's existing clearance check tools or used to inform density overrides of clipped patient anatomy for improved dose calculation accuracy. The system described herein also offers a more cost‐effective approach while achieving this integration, as only an iPad and Structure Sensor or similarly capable hardware are needed to be purchased. Most radiation oncology clinics have existing treatment planning software, and many already have clearance check tools in place. The ClearCheck collision detection utilized in the work presented here is a purchasable product from Radformation, Inc. Although the solution presented needs further refinement to streamline the process, it remains useful as an affordable tool for special cases where additional surface data is beneficial.

It may be observed that the arms and head are included in the CT scans of the five patients included in this study, raising the question of why a 3D surface scan is needed. Currently our clinic follows a protocol to scan these areas to have additional information available for clearance evaluation during treatment planning. Recognizing that all patients in an arms up position were receiving additional CT scan length was a motivating factor to explore ways to avoid this additional imaging dose. An accurate 3D scanned surface model aligned with a CT image set could spare unnecessary radiation dose to healthy organs of future patients. Patient setup variability can alter clearance such that clearance within a few centimeters could still possibly lead to a collision at treatment. Therefore, a warning within 3 cm would likely still be verified at treatment. The results of this study show mean values and standard deviations well within this threshold, and therefore the authors consider this acceptable for clinical use in clearance checks. The sub‐centimeter results are also considered acceptable for adding missing patient surface data for photon volumetric dose calculations. Every clinical case is unique though, and the authors advise to carefully weigh the advantages and risks before implementing these methods.

Considering related studies that have explored 3D surface scanning, Jenkins et al.,^12^ used a Realsense R200 camera, which is a development module that can no longer be purchased commercially. It is similar to the Structure Sensor and that which is currently available on phone and tablet devices and was tested by Jenkins et al on 3D printed phantoms generated from patient CT data. Yoon et al^14^ used LiDAR‐enhanced augmented reality on a handheld device to perform similar work, but no specifics to the hardware are mentioned. The laser scanning method used by Saleh et al^13^ to model a patient surface is complex and not readily accessible for others to implement. Model, point cloud, and mesh evaluations against CT surfaces have shown good results in these prior phantom studies, and our work ventures into direct patient surface scanning analysis. Skinner et al^15^ demonstrated that 3D surface scans of patients with an Intel D415 sensor as well as a Structure Sensor with iPad mini could accurately be used for electron dose computation in head and extremity treatment sites. This supports our study, which expanded into patient thoracic regions and demonstrates 3D scanning accuracy by quantitative evaluation of the model to model distances between each patient's 3D and CT scan generated surfaces. The 3D surface scanning method presented in this study is a simple, low‐cost solution that increases accessibility for a wide range of clinics, including those in low‐resource settings.

Despite the promising results, the current system does have functional limitations. Patient movement may occur between the 3D surface scan and the CT scan and possibly during the 3D surface scan due to the longer acquisition time, which can take up to 1 min. The study results demonstrate limitations in the scans due to clothing and sheets covering the patient, as well as difficulty in scanning all the desired surfaces of the patient due to the user's limited reach. The accuracy of the 3D surface model noticeably declines if the sensor loses track of the scanned area, requiring the user to keep the sensor focused on the area of scanning and preventing the user from walking around the patient when scanning the abdominal region. In contrast, scanning the extremities, thorax, or head is easier, as the user can move around the patient without disrupting the scan. The assistance of a second user or an attachment arm would be critical to have for abdominal and pelvis patients with clipped FOVs in order to fully acquire their surfaces for collision avoidance and calculation depth data. Future studies should explore faster surface scanning methods capable of capturing all angles and be acquired simultaneously with the CT scan ensuring the surfaces acquired from both methods agree.

The 3D surface scanning sensor can have difficulty with infrared light reflection on certain surfaces, such as the patient table. This can be remedied, however, by covering the patient table with a sheet. Although some phantoms may encounter scanning issues due to reflectivity, no issues were seen with the patients’ skin in this study. Excessive skin oils or differing skin tones could potentially play a role in affecting the IR reflectivity. These concerns should be tested further to definitively confirm the impact on 3D scan accuracy. Room brightness may play a role in performance, as excessive light may inhibit detection of infrared reflections, making dim lighting more effective. Standard clinical lighting levels did not present any issues in this study. High gradient surfaces may fail to reflect the infrared back to the sensor, resulting in poor detection. Skin fold areas of the patient typically fall into this category, leading to imperfect conformity of the acquired surface model in these areas.

The pyramid marker used in this study can be replaced with any similarly small object of low density, to thus avoid CT artifacts. The marker may not be critical for alignment if enough anatomical landmarks are present in both the CT images and 3D surface model. A three‐point alignment of markers would be the most helpful, but the markers should be small enough to not be obtrusive, yet large enough to be detected by the 3D surface imaging. BB markers were not used in this work due to concern that they would not be easily discernable in the 3D surface model. More testing of this should be explored. While skin markers in the CT scan may assist in alignment, they may not always fit within the CT field of view (FOV), particularly when a clipped FOV is the reason for surface scan acquisition. The prone patient in this study was a good example of this challenge. Although a portion of the marker was visible and aided alignment, lateral markers are recommended for a prone patient where the back is expected to be clipped on the FOV.

The manual alignment of the 3D surface and the CT scan performed in this study was intended to demonstrate the feasibility of using this surface data for treatment planning with immediately available clinical tools. However, future studies should aim to develop a more automated data alignment method to minimize the discrepancies from potentially inaccurate manual registration. One potential solution is to define a reference space for the 3D surface that is relative to the CT scanner, allowing the 3D surface to be linked to the DICOM coordinates of the CT scan.

Acquiring a 3D surface of a patient may also serve beneficial for mixed reality applications. Augmented and virtual reality are increasingly used for work, games, or everyday tasks. Headsets and eyeglasses with embedded 3D surface scanning sensors have come to market, making these tools more accessible. These technologies offer valuable features that can enhance radiation therapy. Patient surfaces can be converted to holograms which are registered to a treatment machine isocenter and used for a surface‐guided holographic patient setup.^22^, ^23^ Once a 3D surface is incorporated into the treatment planning dataset, it can be used for holographic surface‐guided alignment or any form of surface‐guided radiation therapy.

## 5 CONCLUSION

This study demonstrates the potential of 3D surface scanning to supplement clipped field‐of‐view CT scans and reduce CT scan lengths while obtaining important patient surface data for calculations, treatment clearance checks, and SGRT. While more streamlined solutions are being explored, the methods presented here can be easily implemented as a low‐cost solution to a common radiotherapy problem.

## AUTHOR CONTRIBUTIONS

Amanda M. Jackson: Conceptualization of work, data acquisition, data analysis, data interpretation, draft manuscript preparation, draft manuscript revision, final approval of manuscript; Deepak Shrestha: Data analysis, draft manuscript revision; Perry B. Johnson: Draft manuscript revision, final approval of manuscript

## CONFLICT OF INTEREST STATEMENT

The authors declare no conflicts of interest.

## References

[1] Jones CG , DeCicco J , Sherbini S . Establishment of ALARA. *Health Physics Society* . Accessed November 4, 2025. https://hps.org/publicinformation/ate/q8375/

[2] National Committee on Radiation Protection (US) . Permissible Dose from External Sources of Ionizing Radiation. National Bureau of Standards; 1954:NBS HB 59. doi:10.6028/NBS.HB.59

[3] United States Nuclear Regulatory Commission . Subpart D—Radiation Dose Limits for Individual Members of the Public. *NRC Regulations 10 CFR 20* . Accessed February 24, 2025. https://www.nrc.gov/reading‐rm/doc‐collections/cfr/part020/part020‐1301.html

[4] US Centers for Disease Control and Prevention . Radiation Safety. Radiation and Your Health. February 26, 2024. Accessed February 24, 2025. https://www.cdc.gov/radiation‐health/safety/index.html

[5] Frush DP , Applegate K . Computed tomography and radiation: understanding the issues. J Am Coll Radiol. 2004;1(2):113‐119. doi:10.1016/j.jacr.2003.11.012 17411538

[6] Burke LMB , Bashir MR , Neville AM , Nelson RC , Jaffe TA . Current Opinions on Medical Radiation: a Survey of Oncologists Regarding Radiation Exposure and Dose Reduction in Oncology Patients. J Am Coll Radiol. 2014;11(5):490‐495. doi:10.1016/j.jacr.2013.08.018 24321221

[7] An overview of the key principles of the underlying technologies used by SimRT, MapRT, AlignRT and DoseRT. *Vision RT*. Accessed February 25, 2025. https://visionrt.com/our‐technology/

[8] Wang Z . Review of real‐time three‐dimensional shape measurement techniques. Measurement. 2020;156:107624. doi:10.1016/j.measurement.2020.107624

[9] A Brief Analysis of the Principles of Depth Cameras: Structured Light, TOF, and Stereo Vision. Accessed February 25, 2025. https://wiki.dfrobot.com/brief_analysis_of_camera_principles

[10] Difference between Stereo vision Structured light and ToF. Accessed February 25, 2025. https://www.rfwireless‐world.com/Terminology/Difference‐between‐Stereo‐vision‐vs‐Structured‐light‐vs‐Time‐of‐Flight.html

[11] Baqersad J , Poozesh P , Niezrecki C , Avitabile P . Photogrammetry and optical methods in structural dynamics—A review. Mech Syst Signal Process. 2017;86:17‐34. doi:10.1016/j.ymssp.2016.02.011

[12] Jenkins C , Xing L , Yu A . Using a handheld stereo depth camera to overcome limited field‐of‐view in simulation imaging for radiation therapy treatment planning. Med Phys. 2017;44(5):1857‐1864. doi:10.1002/mp.12207 28295413 PMC5462446

[13] Saleh Z , Pani S , Schmidt M , et al. Feasibility of a Laser Scanning System in Reconstructing 3D Surface for Collision Detection during Radiotherapy. Int J Radiat Oncol Biol Phys. 2024;120(2):e183‐e184. doi:10.1016/j.ijrobp.2024.07.411

[14] Yoon SWW , Zhang Z , Li T . A Feasibility Study of LiDAR‐Enhanced Augmented Reality on a Handheld Device for Collision Detection and Patient Positioning. Int J Radiat Oncol Biol Phys. 2021;111(3):e520‐e521. doi:10.1016/j.ijrobp.2021.07.1423

[15] Skinner L , Knopp R , Wang YC , et al. CT‐less electron radiotherapy simulation and planning with a consumer 3D camera. J Appl Clin Med Phys. 2021;22(7):128‐136. doi:10.1002/acm2.13283 PMC8292688 34042253

[16] Kalantari M , Nechifor M . Accuracy and utility of the Structure Sensor for collecting 3D indoor information. Geo‐Spat Inf Sci. 2016;19:1‐8. doi:10.1080/10095020.2016.1235817

[17] What are the differences between Structure Sensor Pro (ST02B) and Structure Sensor 3 (ST03)?—Structure FAQs. Accessed March 5, 2025. https://support.structure.io/article/453‐what‐are‐the‐differences‐between‐st02b‐and‐st03

[18] Structure—The World's Leading Healthcare 3D Scanning Platform. Accessed March 5, 2025. https://structure.io/structure‐sensor‐3/

[19] Fedorov A , Beichel R , Kalpathy‐Cramer J , et al. 3D Slicer as an Image Computing Platform for the Quantitative Imaging Network. Magn Reson Imaging. 2012;30(9):1323‐1341. doi:10.1016/j.mri.2012.05.001 22770690 PMC3466397

[20] 3D Slicer image computing platform. 3D Slicer. Accessed April 11, 2025. https://slicer.org/

[21] Cometti B . RaySearch and Vision RT unveil automated RayStation®‐MapRT® integration. Vision RT . April 30, 2024. Accessed July 27, 2025. https://visionrt.com/news/raysearch‐and‐vision‐rt‐unveil‐automated‐raystation‐maprt‐integration/

[22] Johnson PB , Jackson A , Saki M , Feldman E , Bradley J . Patient posture correction and alignment using mixed reality visualization and the HoloLens 2. Med Phys. 2022;49(1):15‐22. doi:10.1002/mp.15349 34780068 10.1002/mp.15349

[23] Johnson PB , Bradley J , Lampotang S , et al. First‐in‐human trial using mixed‐reality visualization for patient setup during breast or chest wall radiotherapy. Radiat Oncol Lond Engl. 2024;19(1):163. doi:10.1186/s13014‐024‐02552‐0 10.1186/s13014-024-02552-0 PMC11574990 39558366

